# Extraction and purification of total flavonoids from pine needles of *Cedrus deodara* contribute to anti-tumor *in vitro*

**DOI:** 10.1186/s12906-016-1249-z

**Published:** 2016-07-26

**Authors:** Xiaofeng Shi, Dongyan Liu, Junmin Zhang, Pengbin Hu, Wei Shen, Bin Fan, Quhuan Ma, Xindi Wang

**Affiliations:** 1 Gansu Provincial Academy of Medical Sciences, Lanzhou, 730050 China; 2 Key Laboratory of TCM Pharmacology and Toxicology of Gansu Province, Lanzhou, 730030 China; 3 State Key Laboratory of Applied Organic Chemistry and College of Chemistry and Chemical Engineering, Lanzhou University, Lanzhou, Gansu 730000 China

**Keywords:** Pine needles of *Cedrus deodara*, Total flavonoids, Cancer cell lines, Anti-tumor activity

## Abstract

**Background:**

*Cedrus deodara* is one of the traditional Chinese medicinal herbs that exhibits a line of biological activities. The current study extracted the total flavonoids from the pine needles of *Cedrus deodara* (TFPNCD), and investigated its anti-cancer effects in tumor cell lines.

**Methods:**

The total flavonoids was extracted from pine needles of *Cedrus deodara* by ethanol hot refluxing and purified by HPD722 macroporous resin. The contents of total flavonoids and the active ingredients of TFPNCD were analyzed through UV and HPLC. MTT assay was used to investigate its inhibitory effect on tumor cell lines. The flow cytometry was employed to determine the apoptosis and cell cycle distribution after treated TFPNCD on HepG2 cells.

**Results:**

The TFPNCD, in which the contents of total flavonoid reached up to 54.28 %, and the major ingredients of myricetin, quercetin, kaempferol and isorhamnetin in TFPNCD were 1.89, 2.01, 2.94 and 1.22 mg/g, respectively. The MTT assays demonstrated that TFPNCD inhibited the growth of HepG2 cells in a dose-dependent manner, with the IC_50_ values of 114.12 μg/mL. By comparison, TFPNCD inhibited the proliferation to a less extent in human cervical carcinoma HeLa, gastric cancer MKN28 cells, glioma SHG-44 cells and lung carcinoma A549 than HepG2 cells. We found that even at the lower doses, the total flavonoids effectively inhibited the proliferation of HepG2 cells. Comparison of IC_50_ values implicated that HepG2 cells might be more sensitive to the treatment with total flavonoids. TFPNCD was able to increase the population of HepG2 cells in G0 /G1 phase. Meanwhile, TFPNCD treatment increased the percentage of apoptotic HepG2 cells.

**Conclusion:**

These data suggested that TFPNCD might have therapeutic potential in cancer through the regulation of cell cycle and apoptosis.

## Background

Pinaceae has been shown to comprise eleven genera, of which *Cedrus* (true cedar) was first described by Trew in 1757 [1]. *Cedrus* contains at least four species: *Cedrus deodara* (Roxb.) G. Don, *Cedrus libani* A. Rich., *Cedrus brevifolia* (Hook. f.) Henry and *Cedrus atlantica* (Endl.) Manetti ex Carriére [2, 3]. As one of the widely-used traditional medicine, *Cedrus deodara* displays multiple biological acitivities [4]. In Indian, the wood of *Cedrus deodara* has long been used to treat inflammation and rheumatoid arthritis [5, 6]. In the Dictionary of Chinese Crude Drugs, *Cedrus deodara* has been described to display therapeutic potentials in expelling wind, removing dampness, destroying parasites and relieving itches. Clinically, it is widely used to alleviate arthralgia, traumatic injury, sleeplessness, edema, eczema and acariasis. Recently, the beneficial effects of pine needles have also been reported in patients with rheumatism, cardiovascular diseases, diabetes, obesity, liver and stomach diseases, gonorrhea, chronic bronchitis and cancer.

**Fig. 1:**
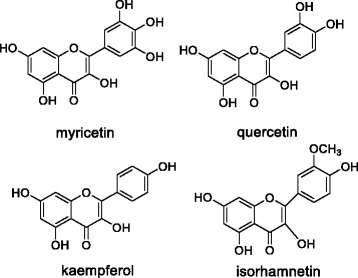
Structures of four major flavonoids in pine needles of *Cedrus deodara*

As an important step to reveal the pharmacological mechanism of *Cedrus deodara*, the chemical studies have successfully extracted three kinds of compounds: terpenes, lignans and flavonoids. As is case for *Cedrus deodara,* these chemical compounds alone exhibit therapeutic activities against pain, spasm, inflammation and cancer. Their antibacterial and antivirus effects have also been reported to date [7]. Previously, our lab has taken great efforts to investigate the chemical constituents of pine needles of *C. deodara*, and has successfully isolated from ethyl acetate extract of pine needles nineteen flavonoids, which include myricetin, quercetin, kaempferol, isorhamnetin and their flavonoid glycosides (Fig. 1) [8, 9]. These four major flavonoids in total flavonoids of pine needles of *C. deodara* (TFPNCD) share similar molecular structures (Fig. 1). Myricetin has been shown to inhibit the proliferation and induce the apoptosis of HepG2 cells probably by means of reducing the phosphorylation levels of several proteins such as Akt, extracellular signal-regulated kinase 1/2 and BAD [10, 11]. Quercetin is also effective in the inhibition of p21-RAS expression in human colon cancer cell lines and in primary colorectal tumors [12].

The current study was designed to extract and purify TFPNCD. We set up the methods to analyze the contents of total flavonoids in TFPNCD as well as the concentrations of myricetin, quercetin, kaempferol and isorhamnetin in TFPNCD. We systematically investigated the effects of TFPNCD on the proliferation of tumor cells *in vitro* and revealed a pronounced change in the cell cycle and apoptosis that was possibly required for TFPNCD to exert anticancer action.

## Methods

### Reagents

3-(4,5-dimethylthiazol-2-yl)-2,5-diphenyltetrazolium bromide (MTT) was purchased from Solarbio Co., Ltd., (Beijin, China). The DEAE Sepharose Fast Flow were obtained from Pharmacia Biotechnology. RPMI-1640 medium was purchased from GIBCO (USA). Fetal bovine serum (FBS) was provided by Sijiqing Corporation (Hangzhou, China). Benzylpenicillin sodium was obtained from Dongfeng Pharmaceutical Co., Ltd., (Jiangxi, China). Streptomycin sulfate was produced by North China Pharmaceutical Group Corporation. The HPLC-grade Acetonitrile was from YuWang Chemical Industry Company (Shan Dong, China). All other chemicals and reagents were of analytical grade. Deionized water was produced with Water Purification system (AFX2-0501-P, Ever Young Enterprises Development Co.LTD, China).

### Plant materials

The pine needles of *Cedrus deodara* (Roxb) G. Don were collected in Lanzhou (Gansu, north of China) in June 2014. To avoid any destruction of chemical components, the collected materials were dried in the shade. The plant sample was identified by Prof. HE Fu-jiang in Gansu Provincial Academy of Medical Science. A fraction of sample was preserved in the plant herbarium of Gansu Provincial Academy of Medical Sciences for future reference.

### Preparation of TFPNCD

The powder (40 mesh) of pine needles of *Cedrus deodara* (30 g) was extracted two times (2 h and 1 h) with 40 % ethanol (25 times volume) and then filtered. The filtrate was evaporated under reduced pressure by using a rotary evaporator (BUCHI, Switzerland) to obtain the crude extract solution, with the concentration of 2.85 mg/mL. The crude extract solution (15 mL; adjusted to pH 4.0) was loaded in glass columns which were wet-packed with HPD722 macroporous resin (10 g). The feed rate was set at 2 bed volume (BV) · h^−1^. The adsorbate-laden column was washed with 4 BVof deionized water, and then eluted with 2 BV of aqueous solution (70 % ethanol) at 2 BV · h^−1^. The eluting solution was concentrated in the rotary evaporation apparatus and dried under vacuum before further analysis. All the dynamic experiments were performed at room temperature.

### Measurement of the total flavonoids content in TFPNCD

The total flavonoids in TFPNCD were estimated as rutin equivalent. To draw the calibration curve, a series of rutin solutions (concentration range: 4.58–54.9 μg/mL) were prepared as below: the appropriate volumes of rutin stock solution were diluted with 0.3 mL of 5 % (w/v) NaNO_2_, 0.3 mL of 10 % Al(NO_3_)_3_ and 4 mL of 4 % (w/v) NaOH. The mixtures were allowed to stand for 6 min before diluted to final total 25 mL with distilled water. Fifteen min later, the absorbance was measured with a spectrometer (Shimadzu Co., Ltd, Japan). The TFPNCD sample was prepared and analyzed as above. The total flavonoids content of TFPNCD was calculated based on the established calibration curve.

### Measurement of four flavonoids contents in TFPNCD

TFPNCD was analyzed by Agilent 1100 HPLC TC-C_18_ column (150 mm × 4.6 mm; 5 μm, USA) equipped with UV-detector system. The mobile phase was methanol(A)-water(B) with the gradient elution (0–7 min, 55 % A; 7–20 min, 80 % A; 20–25 min, 55 % A) at the flow rate of 1.0 mL/min at 30 °C and the detection was performed at 360 nm wavelength. The sample pre-treatment process involved the reflux extraction with 60 % methanol for 1 h and with the ratio of liquor to material of 25 mL/g, extracting solution hydrolysis with 10 % hydrochloric acid for 1 h. All tested solutions were of spectra analytical grade and were filtered through 0.45 μm filters before use. The standard of myricetin was purchased from Ronghe medical technology Co. Ltd (Shanghai, China), while those of quercetin, kaempferol and isorhamnetin were purchased from Chinese food and Drug Inspection Institute (Beijing, China).

### Cell culture

The Human Cervical Carcinoma HeLa Cells, gastric cancer MKN28 cells, human lung carcinoma A549 Cells, human hepatocellular carcinoma HepG2 cells and human glioma SHG-44 cells were purchased from the Cell Bank of Shanghai Institute of Cell Biology, Chinese Academy of Sciences (Shanghai, China). These cells were cultured in RPMI-1640 medium (HyClone, USA), except for HepG2 in DMEM medium (HyClone, USA). Both culture media was were supplemented with 10 % fetal bovine serum (Gibco, Australia), 50 U/mL each of penicillin and streptomycin and incubated at 37 °C in a humidified incubator containing 5 % CO_2_ in air. The medium was changed twice a week.

### Cell growth inhibition

Cancer cells at logarithmic growth phase were digested by 0.25 % trypsin, adjusted to the concentration of 2 × 10^4^ cells/mL, inoculated into 96-well cell culture plates (200 μL per well) and then cultured at 37 °C for 24 h. After treatment with various concentrations of TFPNCD (5, 10, 20, 30, 40, 50, 60, 80, 100 μg /mL) for 44 h, the cell proliferation was measured by MTT assay according to the manufacturer’s instructions. Briefly, 20 μL of MTT reagent (5 mg/mL) was added to each cluster well and incubated at 37 °C for 4 h. The medium with MTT was then removed and 150 μL of DMSO was added to solubilize the formazan crystals. The plates were agitated gently for 6 min at room temperature. The absorbance value per well was determined at 570 nm using a xMark microplate reader (BioRad Co., USA). All assays were repeated three times. The cell proliferation inhibition rate (%) was calculated as follows: Cell proliferation inhibition rate (%) = (1 - mean OD value / control mean OD value) × 100.

### Cell cycle analysis

The human hepatoma HepG2 cells during logarithmic growth phase were routinely cultured with DMEM. After digestion with 0.25 % trypsin, 5 × 10^5^/mL cells were inoculated into 6-well cluster and cultured at 37 °C overnight. The cells were treated with different concentrations of TFPNCD (10, 20, 40, 80, 160 μg/mL) for 48 h before digestion with trypsin. The cell suspension was centrifuged for 5 min at 1000 rpm/min. The cell pellet was washed three times with cold phosphate-buffered saline (PBS), fixed with 70 % cold ethanol at 4 °C for 24 h, and then supplemented with 100 μL of 100 μg/mL RNaseA for 30 min at temperature room. After two washes with PBS, the cells were stained with 400 μL of 100 μg/mL PI and incubated in the dark for more than 5 min. The samples were placed in 12 × 75 Falcon tubes and evaluated by a FACS Calibur flow cytometry (Becton-Dickinson, USA).

### Apoptosis measurement

An Annexin V-FITC/PI double staining method was carried out according to the manufacturer’s instruction. Human hepatoma HepG2 in logarithmic growth phase were seeded in 6-well culture clusters. After 48 h treatment with different doses of TFPNCD, the cells were digested with trypsin and collected. The cells were washed two times with cold PBS and centrifuged for 5 min at 1000 rpm/min. The adherent and floating cells were suspended in 400 μL of Binding Buffer with the concentration of 5 × 10^5^/mL. The cells were incubated with 5 μL annexin V-FITC and 5 μL PI in the dark for 15 min at room temperature, and the apoptotic cells were analyzed by a FACS Calibur flow cytometry (Becton-Dickinson, USA).

### Statistical analysis

Statistical analysis was conducted by using SPSS 16.0 software. All data were presented as mean ± standard deviation values. Statistical differences between groups were analyzed by one-way analysis of variance (ANOVA) and Student’s t-test with statistical significance set at *p* < 0.05 or *p* < 0.01.

## Results

### The content of total flavonoids in TFPNCD

The concentration of flavonoids was calculated by using rutin as the calibration standard. A good linear relationship was obtained when flavonoid contents ranged from 4.58 μg/mL to 54.9 μg/mL. The resultant regression equation was as follows: y = 0.0012 x + 0.0048 (*R* = 0.9993), where y, x and R represented the absorbance at 510 nm, the concentration of flavonoids (mg/mL) and the regression coefficient, respectively. The average recovery was 97.64 % ± 1.31 % (*n* = 6). By using this method, we found that the content of total flavonoid in TFPNCD was about 54.28 %.

### The contents of four flavonoids in TFPNCD

**Fig. 2:**
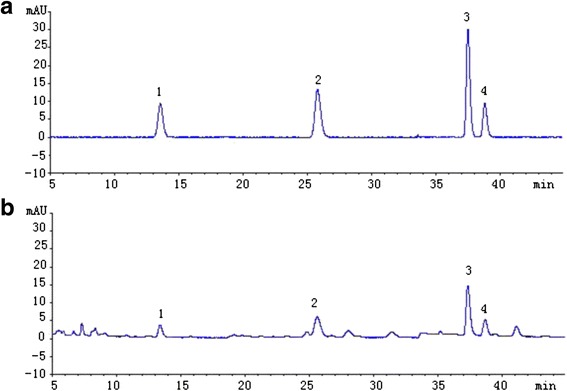
HPLC chromatograms of reference substances (**a**) and sample (**b**). 1. myricetin; 2. quercetin; 3. kaempferol; 4. isorhamnetin

Myricetin, quercetin, isorhamnetin and kaempferol have been identified as the marker components in TFPNCD. We therefore determined their contents through the standard method as described above. The calibration curves were linear when the concentrations of myricetin, quercetin and isorhamnetin were adjusted to 1 ~ 10 μg/mL. A good linear curve was also obtained with kaempferol at the concentration of 4 ~ 40 μg/mL (r ≥ 0.9992). The regression equations for the four compounds were summarized in Table 1. In addition to the satisfactory reproducibility, the time course required to measure the contents of myricetin, quercetin, kaempferol and isorhamnetin in TFPNCD was as short as 14.6, 27.2, 38.3 and 39.6 min (Fig. 2). Our data showed that the contents of myricetin, quercetin, kaempferol and isorhamnetin in TFPNCD were 1.89, 2.01, 2.94 and 1.22 mg/g, while their average recoveries were 100.14, 97.63, 102.11 and 100.45 %, respectively (*n* = 6).

**Table 1:** Linear relations

Compound	Regression equation	*r*	Linear range/μg · mL^-1^
myricetin	*Y* = 47.087*X* -11.842	0.9993	1 ~ 10
quercetin	*Y* = 46.326*X* -11.270	0.9993	1 ~ 10
kaempferol	*Y* = 58.578*X* -51.830	0.9993	4 ~ 40
isorhamnetin	*Y* = 41.387*X* -9.354	0.9992	1 ~ 10

### Anti-proliferative effects of TFPNCD in HepG2 cells

**Fig. 3:**
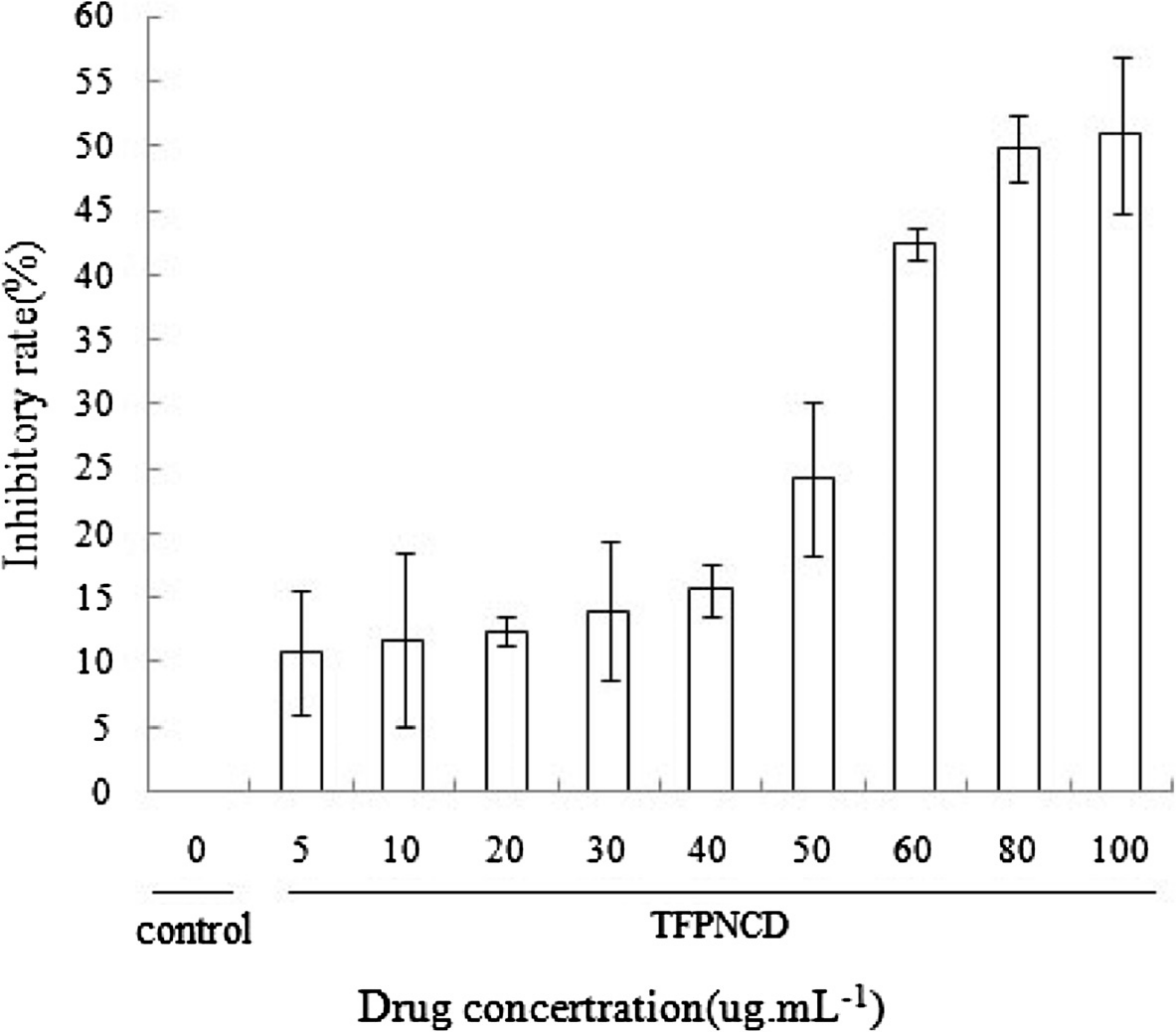
Anti-proliferative effects of TFPNCD on hepatocellular carcinoma HepG2 cells. Cells were treated with different doses of TFPNCD (5, 10, 20, 30, 40, 50, 60, 80, 100 μg /mL) for 48 h before the measure of cell proliferation by MTT assay. Data were expressed as mean ± SD deviation values obtained from three independent determinations. **P < 0.01 versus controls

To observe the possible influence of TFPNCD on cell proliferation, we treated the human cervical carcinoma HeLa Cells, gastric cancer MKN28 cells, lung carcinoma A549 Cells, hepatocellular carcinoma HepG2 cells and glioma SHG-44 cells with different doses of TFPNCD for 48 h before MTT assays. Our data demonstrated that TFPNCD (5–100 μg/mL) inhibited the growth of HepG2 cells in a dose-dependent manner (Fig. 3). The IC_50_ values were 114.12 μg/mL, suggesting that TFPNCD displayed a more potent inhibition of HepG2 cells. By comparison, TFPNCD inhibited the proliferation to a less extent in human cervical carcinoma HeLa, gastric cancer MKN28 cells, glioma SHG-44 cells and lung carcinoma A549 than HepG2 cells.

### Effects of TFPNCD on cell cycle

**Fig. 4:**
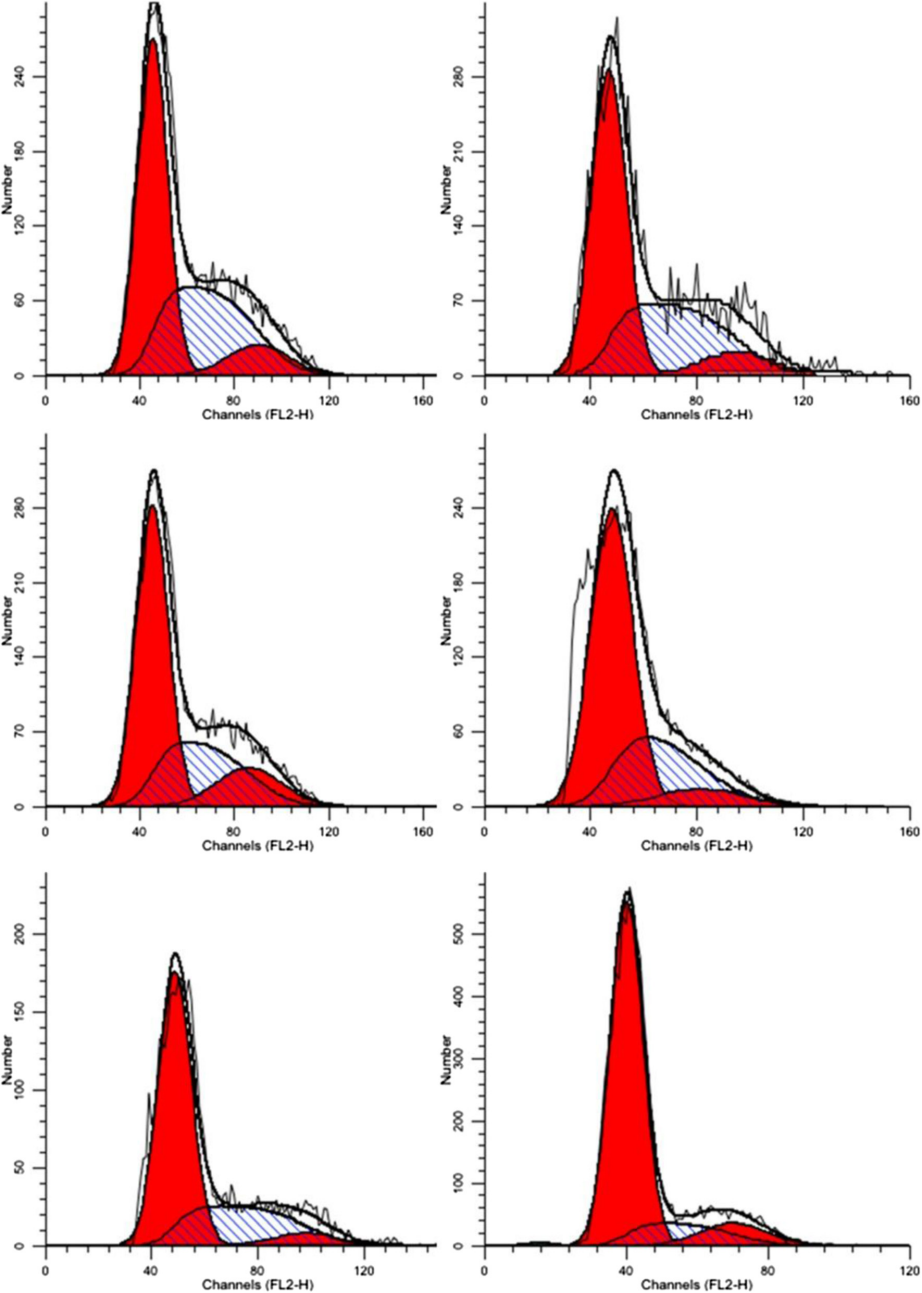
Effects of TFPNCD on the cycle distribution of HepG2 cells. The cells were treated with different doses of TFPNCD (10, 20, 40, 80, 160 μg/mL) for 48 h. Cells were washed, fixed and stained with propidium iodide (PI) before Becton-Dickinson FACS Calibur flow cytometry analysis

Next, we investigated the potential effects of TFPNCD on cell cycle. To this end, the HepG2 cells were incubated with different doses of TFPNCD for 48 h, followed by flow cytometric analysis. Our data showed that, with the increase of TFPNCD doses from 10 to 160 μg/mL, the percentage of cells in G0 /G1 phase was also gradually increased. At 160 μg/mL, TFPNCD enhanced the cell population at G0 /G1 phase to 76.67 ± 2.87 % relative to 51.72 ± 1.26 % in mock-treated control cells (Fig. 4). The percentages of cells at different cell cycle phases after TFPNCD treatment were summarized in Table 2.

**Table 2:** Percentages of HepG2 cells in G0/G1-, S-, and G2/M-phase (±s,*n* = 3)

Group	Percentages (%)		
	G_0_/G_1_	S	G_2_/M
0 (control)	51.72 ± 1.26	39.03 ± 1.14	9.25 ± 0.87
10 μg/mL	53.96 ± 1.34	37.86 ± 1.89	8.18 ± 2.05
20 μg/mL	55.84 ± 0.98	29.99 ± 2.01^*^	14.17 ± 0.83^*^
40 μg/mL	63.55 ± 1.65^*^	29.13 ± 1.02^*^	7.32 ± 1.64
80 μg/mL	64.61 ± 1.32^*^	29.81 ± 0.78^*^	5.58 ± 0.12^*^
160 μg/mL	76.67 ± 2.87^**^	13.38 ± 1.26^**^	9.95 ± 0.67

*P < 0.05,**P < 0.01 versus controls

### Apoptosis effect of TFPNCD on HepG2 cells

**Fig. 5:**
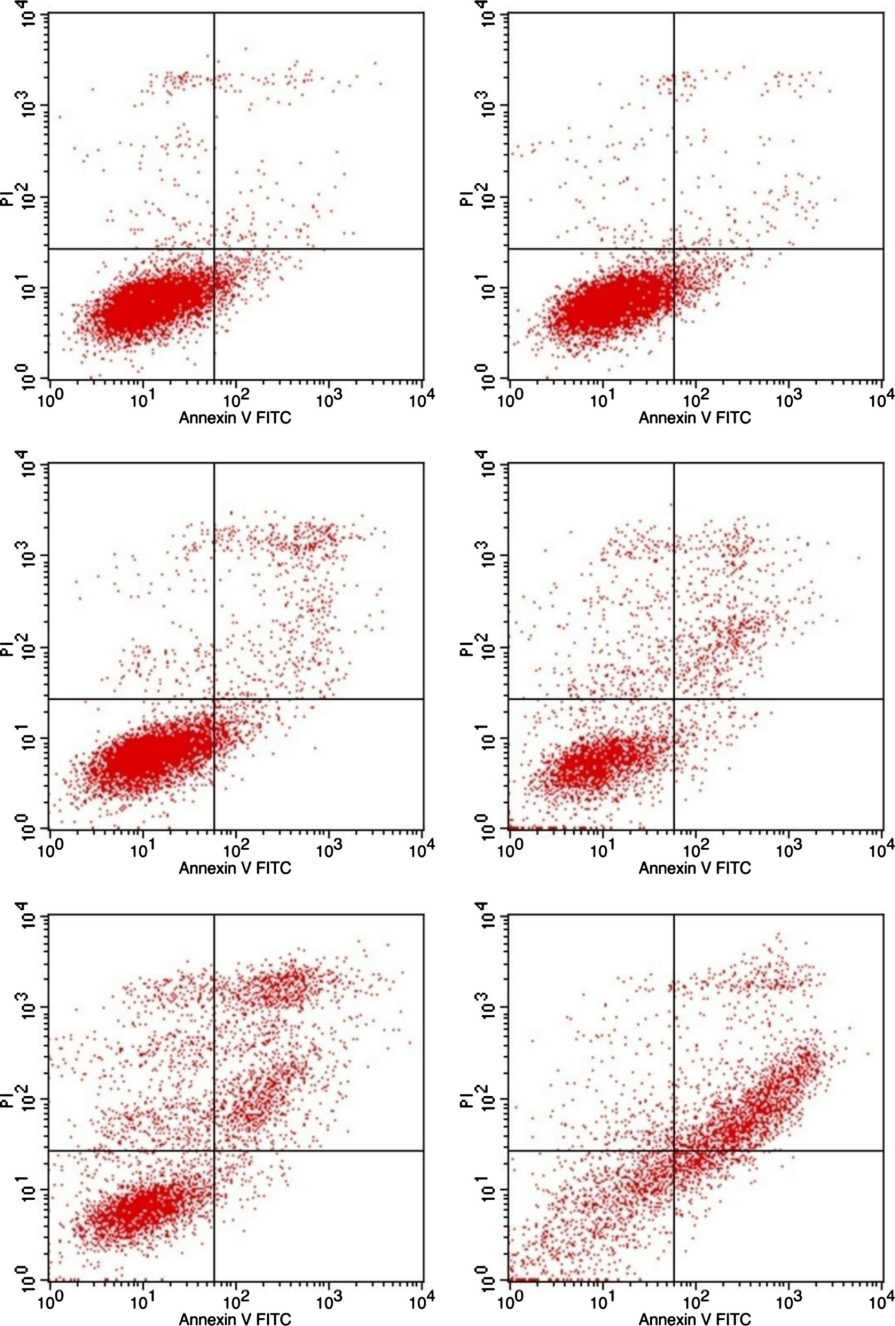
Apoptotic effect of TFPNCD treatment in HepG2 cells. The cells were treated with different doses of TFPNCD (10, 20, 40, 80, 160 μg/mL) for 48 h and then stained with annexin V-FITC and propidium iodide (PI) as described in Materials and Methods. The stained cells were analyzed for apoptosis by using a Becton-Dickinson FACS Calibur flow cytometry

Our data showed that the HepG2 cells in the lower left quadrant were viable and negative for both annexin V and PI. The early apoptotic cells in the upper left quadrant were positive for annexin V but negative for PI. The late apoptotic cells in the upper right quadrant were positive for annexin V and PI. The necrotic cell population in the lower right quadrant was positive for PI and negative for annexin V (Fig. 5). Quantitative analysis demonstrated that the amount of apoptotic HepG-2 cells (both early and late apoptosis) significantly increased after exposure to TFPNCD for 48 h. As shown in Table 3, TFPNCD at the doses of 20, 40, 80, 160 μg/mL increased the apoptotic ratea from 6.44 ± 0.97 % of control to 12.71 ± 1.29 % (*n* = 3, *p* < 0.05), 20.53 ± 2.07 % (*n* = 3, *p* < 0.01), 33.86 ± 1.17 % (*n* = 3, *p* < 0.01) and 64.56 ± 2.05 % (*n* = 3, *p* < 0.01), respectively.

**Table 3:** The influence of flavonoids of *Cedrus deodara* (Roxb.) Loud. pine needle on HepG2 tumor cell apoptosis $\left({}_{\overline{x}}\pm s,n=3\right)$

Group	Cell apoptosis distribution percentage and apoptosis rate				
	UL (%)	UR (%)	LL (%)	LR (%)	Apoptosis (%)
0 (control)	2.16 ± 0.13	1.78 ± 0.34	91.40 ± 1.83	4.66 ± 0.57	6.44 ± 0.97
10 μg/mL	1.57 ± 0.08	2.10 ± 0.23	91.25 ± 2.27	5.08 ± 0.61	7.18 ± 0.83
20 μg/mL	2.04 ± 0.12	8.05 ± 1.96^*^	85.25 ± 2.79^*^	4.66 ± 0.41	12.71 ± 1.29^*^
40 μg/mL	9.28 ± 1.76^*^	16.98 ± 0.72^**^	70.18 ± 1.24^**^	3.55 ± 0.56	20.53 ± 2.07^**^
80 μg/mL	14.62 ± 1.02^**^	31.68 ± 1.45^**^	51.51 ± 1.38^**^	2.18 ± 0.17	33.86 ± 1.17^**^
160 μg/mL	6.16 ± 0.66^**^	52.66 ± 2.82^**^	29.28 ± 1.65^**^	11.90 ± 1.17^**^	64.56 ± 2.05^**^

*P < 0.05,**P < 0.01 versus control

## Discussion

Great efforts have to date been made to develop the anti-cancer drugs. The natural products are attracting increasing attention due to their many advantages over chemical drugs, such as the low toxicity, safety and cheapness [13]. In 1963, a crude extract from the bark of the Pacific yew Taxus brevifolia, a kind of scarce evergreen that grows slowly in the old-growth forest of Pacific Northwest, was found in preclinical studies to have cytotoxic activity against many tumors [14]. Since then, a diterpenoid compound, paclitaxel, was successfully isolated from the bark of the Taxus brevifolia. Now paclitaxel has been substantiated to be the major component that exerts anti-cancer effect. In 1992, paclitaxel was approved by the Food and Drug Administration as one of the most important chemotherapeutic agents against a wide range of tumors [15]. Currently, a series of paclitaxel analogues have been discovered and synthesized to treat the patients with advanced cancer of the ovaries, breast, lung, and kaposi sarcoma.

*Cedrus deodara* has been shown to contain the non-volatile and volatile constituents. Most of non-volatile constituents include terpenes, lignans, flavonoids and fatty acids [16]. By using spectroscopic methods such as ^1^H NMR, ^13^C NMR, IR, and LC-MS, previous studies have isolated from the dried heartwood powder of *Cedrus deodara* three compounds: (−)-matairesinol, (−)-nortrachelogenin, and a dibenzylbutyrolactollignan (4, 4’, 9-trihydroxy-3, 3’-dimethoxy-9, 9’-epoxylignan). Three compounds have been revealed to display potent antioxidant activities. Thereafter, two lignans were discovered in lead acetate-purified butanol soluble fraction of wood of *Cedrus deodara* [17]. The phytochemical screening of its leaf part further shows the presence o flavanoids, alkaloids, tannins and saponins [18]. Recently, an isolated “CD lignan mixture” from stem wood of the plant has been shown to contain high concentrations of (−)-wikstromal (75–79 %), (−)-matairesinol (9–13 %) and benzylbutyrolactol (7–11 %). The *in vitro* assays demonstrate that this mixture exhibits the pronounced anticancer activities. More importantly, the pharmacological studies in animal models of Ehrlich ascites carcinoma and colon carcinoma (CA-51) have also consolidated its dose-dependent action against several cancer cell lines such as cervix, colon, liver, prostate and neuroblastoma *in vivo* [19–21]. The lignan mixture might exert anti-cancer effects by regulating annexin V binding, intracellular caspases activities and DNA fragmentation [22–24].

Flavonoids are ubiquitously distributed in nature. As the food-derived cancer preventive compounds, they display potential therapeutic benefit in cancer and may be considered as the candidates for chemotherapeutic agents [25]. Investigation of the clinical use of polyphenolic compounds, such as quercetin, kaempferol, apigenin, myricetin, baicalein, luteolin, isorhamnetin, chrysin, nobiletin, and tangeretin, might be valuable strategies in the development of novel anticancer drugs [26–29]. To improve their biological activities and enhance their anticancer effects, it is also important to chemically modify the structures of natural flavonoids by introducing structural variations into their backbone. The current study purified and extracted the total flavonoids from pine needles of *Cedrus deodara*. After successful measurement of the content of total flavonoids in TFPNCD through HPLC techniques, we systematically evaluated the biological activity of total flavonoids against tumor cells. Our data showed that even at the lower doses, the total flavonoids effectively inhibited the proliferation of HepG2 cells. Comparison of IC_50_ values implicated that HepG2 cells might be more sensitive to the treatment with total flavonoids.

Dysregulation of apoptosis is one of the key mechanisms underlying cancer [30]. Activation of apoptotic pathways in cancer cells is thus critical for cancer therapy. The means by which an herbal product induces apoptosis has become the subject of great interest. In this study, we revealed that total flavonoids exhibited potent cytotoxicity against HepG2 cells. To gain insight into the mechanisms underlying the anti-cancer effects of total flavonoids, we investigated the possible changes in the cell cycle and apoptosis. We found that the total Flavonoids was able to retard the HepG2 cells at G0 /G1 phase. Consistent with these results, the apoptotic rate of HepG2 cells was also increased by the total flavonoids in a dose-dependent way. These data broadened our framework for the understanding of the medicinal function of *Cedrus deodara,* and provided evidence for the first time that the total flavonoids exerted a potent inhibitory effect on the tumor growth. Especially, the TFPNCD might have therapeutic potential in cancer by regulating cell cycle and apoptosis. The anti-cancer action of the total flavonoids *in vivo*, however, requires further studies in the future.

## Conclusions

In summary, the present study demonstrates that the total flavonoids of *Cedrus deodara* were able to to inhibit the tumor proliferation. The anticancer action of TFPNCD might involve the regulation of cell cycle and apoptosis. An important step toward a better evaluation of TFPNCD is to extensively study how this flavonoids act in *vivo* in the future.

## Abbreviations

BV, bed volume; FBS, fetal bovine serum; MTT, 3-(4,5-dimethylthiazol-2-yl)-2,5-diphenyltetrazolium bromide; TFPNCD, total flavonoids from pine needles of Cedrus deodara
